# A systematic evaluation and meta-analysis of early prediction of post-thrombotic syndrome

**DOI:** 10.3389/fcvm.2023.1250480

**Published:** 2023-08-24

**Authors:** Tong Yu, Jialin Song, LingKe Yu, Wanlin Deng

**Affiliations:** ^1^ Pharmacy Laboratory, College of Pharmacy, Shenyang Pharmaceutical University, Benxi, China; ^2^ Microbiology laboratory, College of Life Sciences and Pharmacy, Shenyang Pharmaceutical University, Benxi, China; ^3^ Department of Encephalopathy, Internal Medicine Department, Liaoning University of Traditional Chinese Medicine Affiliated Second Hospital, Shenyang, China; ^4^ Electrical Engineering, Information Engineering College, Shenyang University of Chemical Technology, Shenyang, China

**Keywords:** post-thrombotic syndrome, venous thrombosis, model prediction, meta-analysis, prospective cohort study

## Abstract

**Objective:**

Post-thrombotic syndrome (PTS) is the most common long-term complication in patients with deep venous thrombosis, and the prevention of PTS remains a major challenge in clinical practice. Some studies have explored early predictors and constructed corresponding prediction models, whereas their specific application and predictive value are controversial. Therefore, we conducted this systematic evaluation and meta-analysis to investigate the incidence of PTS and the feasibility of early prediction.

**Methods:**

We systematically searched databases of PubMed, Embase, Cochrane and Web of Science up to April 7, 2023. Newcastle-Ottawa Scale (NOS) was used to evaluate the quality of the included articles, and the OR values of the predictors in multi-factor logistic regression were pooled to assess whether they could be used as effective independent predictors.

**Results:**

We systematically included 20 articles involving 8,512 subjects, with a predominant onset of PTS between 6 and 72 months, with a 2-year incidence of 37.5% (95% CI: 27.8–47.7%). The results for the early predictors were as follows: old age OR = 1.840 (95% CI: 1.410–2.402), obesity or overweight OR = 1.721 (95% CI: 1.245–2.378), proximal deep vein thrombosis OR = 2.335 (95% CI: 1.855–2.938), history of venous thromboembolism OR = 3.593 (95% CI: 1.738–7.240), history of smoking OR = 2.051 (95% CI: 1.305–3.224), varicose veins OR = 2.405 (95% CI: 1.344–4.304), and baseline Villalta score OR = 1.095(95% CI: 1.056–1.135). Meanwhile, gender, unprovoked DVT and insufficient anticoagulation were not independent predictors. Seven studies constructed risk prediction models. In the training set, the c-index of the prediction models was 0.77 (95% CI: 0.74–0.80) with a sensitivity of 0.75 (95% CI: 0.68–0.81) and specificity of 0.69 (95% CI: 0.60–0.77). In the validation set, the c-index, sensitivity and specificity of the prediction models were 0.74(95% CI: 0.69–0.79), 0.71(95% CI: 0.64–0.78) and 0.72(95% CI: 0.67–0.76), respectively.

**Conclusions:**

With a high incidence after venous thrombosis, PTS is a complication that cannot be ignored in patients with venous thrombosis. Risk prediction scoring based on early model construction is a feasible option, which helps to identify the patient's condition and develop an individualized prevention program to reduce the risk of PTS.

## 1. Introduction

Post-thrombotic syndrome (PTS) is a chronic and common complication of deep vein thrombosis (DVT) (1), mainly caused by damage to the blood vessel wall and the obstruction of venous return due to venous thrombosis (2). The main symptoms of PTS include edema, lower extremity pain, pigmentation changes, skin pruritus, ulcers, etc., but early DVT is usually asymptomatic, resulting in inadequate or delayed diagnosis due to a lack of awareness of risk factors and symptoms. Meanwhile, PTS can cause severe disability and impaired quality of life and require significant health care costs (1, 3).

Global, multicenter epidemiological data on PTS are lacking, but studies have shown that the cumulative incidence of PTS plateaus 1–2 years after onset and that the incidence of severe PTS, including venous ulcers, continues to accumulate, with up to one-third of patients developing the disease at 6 years (4). A study (5) showed that in the United States National Hospital Discharge Survey during 2007–2009, annual VTE-related hospitalizations involved 547,596 adult patients out of a population of 307 million. At the same time, the pathophysiological changes in PTS include a complex series of processes, and endoluminal treatment alone cannot resolve all of the patient's signs and symptoms, and the recurrence rate after surgery is extremely high. Therefore, improving the level of DVT prevention and treatment is essential to reduce PTS.

With the rapid development of the medical environment and medical technology, systematic knowledge of the incidence of PTS can help develop prevention and control strategies for the disease in clinical practice. There is currently a lack of evidence-based proof and comprehensive understanding of the onset and early predictors of PTS, as well as a lack of clinically validated risk prediction tools for the early prediction of PTS, which poses a serious challenge to the clinical prevention and management of PTS (6). In this context, researchers have started to explore valid predictors and attempted to construct risk assessment tools based on these valid predictors, while the predictive value and predictors of these risk assessment tools are controversial. Hence, we conducted this systematic evaluation and meta-analysis to describe disease incidence from an evidence-based perspective and to comprehensively summarize the predictive value of effective independent predictors and prediction models constructed based on predictors, in order to provide a valuable reference for the evaluation of subsequent risk prediction models.

## 2. Methods

### 2.1. Study registration

The present study was conducted in accordance with the Preferred Reporting Items for Systematic Reviews and Meta-Analyses (PRISMA 2020) and prospectively registered on PROSPERO (ID: CRD42023417848).

### 2.2. Eligibility criteria

**Inclusion criteria:**

**Figure 1:**
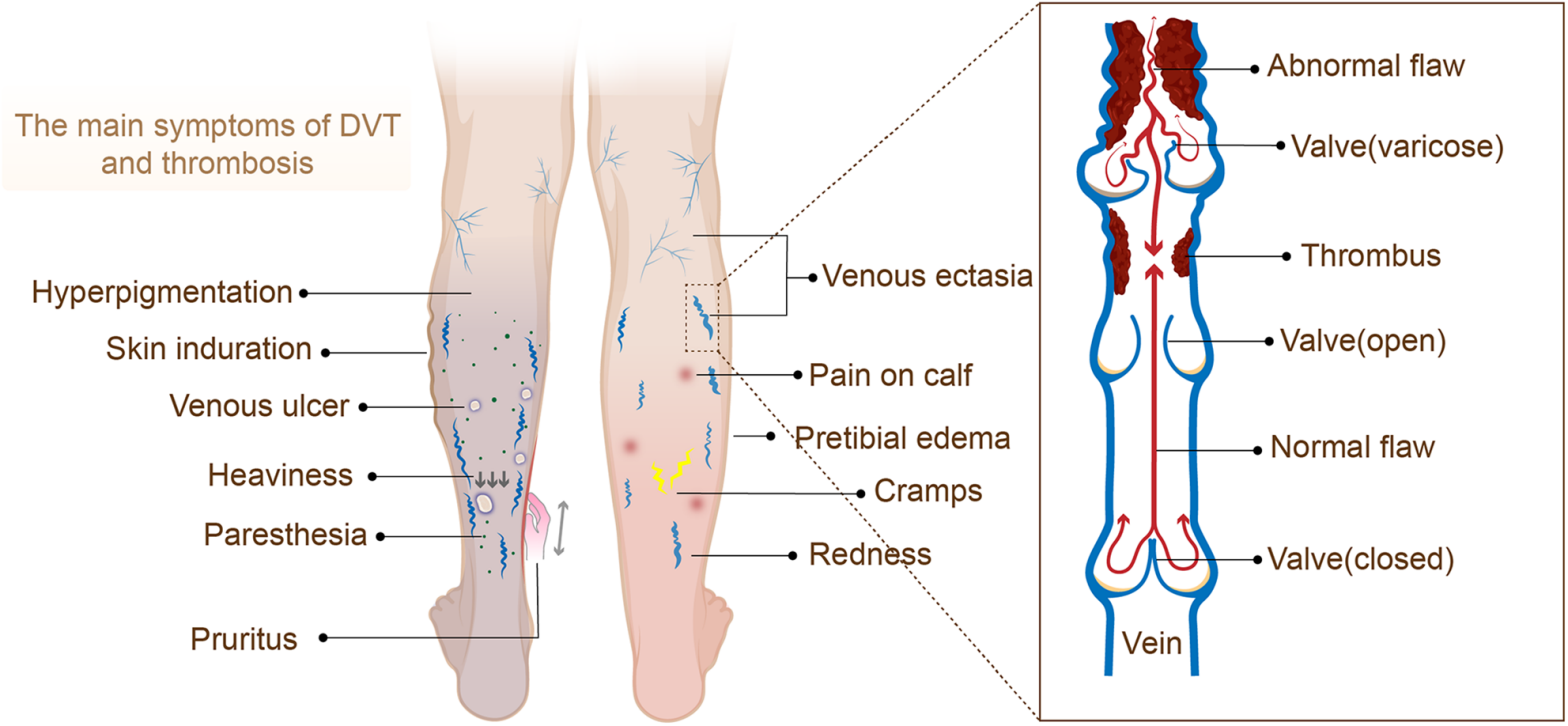
The main symptoms of DVT and thrombosis.

**Figure 2:**
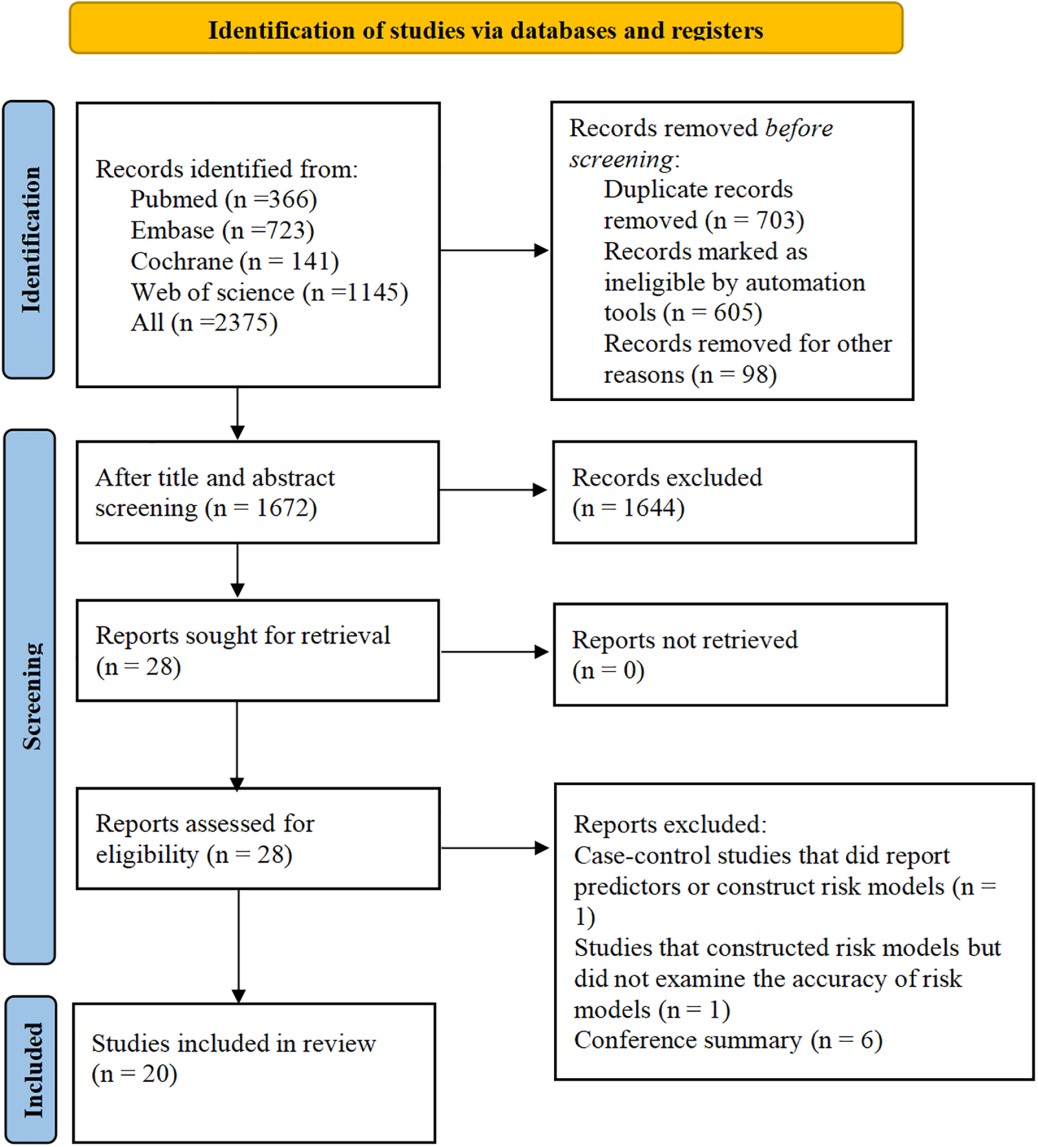
Literature selection process.

(1) Patients with confirmed deep venous thrombosis were included in the study (Figure 1);
(2) The study types were RCTs, case-control studies, cohort studies, and nested case-control studies;
(3) The prediction model of PTS was completely constructed or the risk factor analysis of PTS was conducted;
(4) In the research of prediction model, the studies without external validation were also included in this systematic review;
(5) Research on different types of machine learning published on the same dataset;
(6) Included literature reported in English.

**Exclusion criteria:**

(7) The research types were meta-analyses, reviews, guidelines, expert opinions, etc.;
(8) Risk factor analysis was not performed and a complete machine learning model was not constructed;
(9) Studies with rather small sample sizes (<50 cases);
(10) Validation studies of mature scales alone;

### 2.3. Data sources and search strategy

We systematically searched PubMed, Embase, Cochrane and Web of Science databases until 7 April 2023. The search was conducted in the form of subject terms and free words, with no restrictions on regions. The detailed search strategies are shown in Supplementary Material S1.

### 2.4. Study selection and data extraction

In this systematic review, EndNote was first used to de-duplicate the retrieved literature, and then we initially screened eligible original studies by reading the titles and abstracts and downloaded their full text. Next, we further read the full text to screen the original studies that met the requirements of our review.

Before data extraction, a standardized spreadsheet was used to develop a data extraction plan. The extraction information included title, first author, publication year, author's country, study type, patient source, background disease, diagnostic criteria for post-thrombotic syndrome, follow-up duration, number of post-thrombotic syndrome cases, the total number of cases, number of post-thrombotic syndrome cases in the training set, the total number of cases in the training set, generation method of the validation set, overfitting method, number of post-thrombotic syndrome cases in the validation set, the total number of cases in the validation set, missing value processing method, variable screening, type of model used, modeling variables.

The above literature screening was carried out by two investigators (Tong Yu and Jialin Song). After the completion of cross-checking, the third researcher (Lingke Yu) was asked to assist in adjudicating if there was any dispute.

### 2.5. Assessment of study quality

The original types of studies included in our systematic review were cohort or case-control studies, and thus we adopted the Newcastle-Ottawa Scale (NOS) to evaluate the quality of the included original studies (7). The variable applies to cohort studies and pathologically controlled studies, and the quality evaluation is mainly conducted from eight questions in three fields. Except for the full score of 2 points for comparability, the full score of the remaining seven questions is 1 point. A score of 7 to 9 was considered a high-quality study and a score of 4 to 7 was defined as a moderate-quality study.

For this study, two evaluators (Tong Yu and Jialin Song) performed a risk of bias assessment using NOS and cross-checked. If there was any discrepancy in the evaluation, the third evaluator (Wanlin Deng) was invited to participate in the decision.

### 2.6. Outcomes

In this systematic review, we pooled the incidence rates in cohort studies. The incidence of PTS was calculated as the percentage of subjects with PTS in each cohort study. The odds ratio (OR) of each predictor was derived from multivariate logistic/cox regression or ORs adjusted for other confounders. The c-index, sensitivity, and specificity of the prediction model constructed based on these predictors were used to reflect its overall accuracy. The primary outcomes encompassed the incidence of PTS and the c-index, sensitivity, and specificity of the prediction model. The secondary outcome was the ORs of predictors under multivariate analysis.

### 2.7. Synthesis methods

When performing independent predictor analyses, to avoid the influence of confounding factors, we extracted ORs from multivariate logistic/cox regressions from original studies or those adjusted for other confounding factors for meta-analysis. A random-effects model was used when the heterogeneity index *I*^2^ > 50% in the meta-analysis of the OR of the predictors and subgroup analysis was performed according to the different levels of each predictor.

In addition, our outcome measures included indicators to evaluate the accuracy of the machine learning model (c-index, sensitivity, and specificity). When evaluating the c-index, we prioritized the use of a random-effects model for data processing due to differences in the inclusion of variables and inconsistent parameters across machine-learning models.

For the c-index with missing 95% confidence intervals and standard errors, we referred to the study of Debray TP et al. (8) and estimated the standard errors. Also, we performed a meta-analysis of sensitivity and specificity using a bivariate mixed-effects model. The meta-analysis of this study was conducted based on R4.2.0 (R development Core Team, Vienna, http://www.R-project.org).

## 3. Results

### 3.1. Study selection

A total of 2,375 papers were retrieved, 603 of which were duplicates, and 28 eligible studies were included according to the titles and abstracts. Twenty studies (9–28) were retained after full-text review. The literature screening process is shown in Figure 2.

### 3.2. Study characteristics

The present study included 20 studies altogether, with a total number of 8,512 cases and 3,219 cases of morbidities. The period of publication is mainly from 2016 to 2023. The countries of publication included China (9, 11, 12, 18, 21), Norway (10, 28), Canada (13), the Netherlands (14, 16, 25), Japan (15), Indonesia (17), Australia (19), Switzerland (20), Israel (22, 23), Pakistan (24) and Poland (26, 27). For the background of morbidity, only one of the studies (10) reported a background of pregnancy-related venous thrombosis, while the remaining 19 had a background of deep vein thrombosis (DVT). As for the diagnostic criteria of PTS, two studies (14, 15) used the CEAP score (CEAP score ≥3) and the remaining 18 used the Villalta score (Villalta score ≥5). Among the 18 studies adopting the Villalta score, four used a new scale developed on the basis of the Villalta score to assess PTS, one (18) employed the Villalta score combined with the APTSD score, two (22, 23) used the Villalta score with SOX-PTS score, and one (16) used Villalta score and LET grading score. The follow-up period of the articles ranged from 3 months to 72 months. Seven articles (9, 11, 12, 18, 21, 25) constructed risk prediction tools with independent validation sets, with a total of 21 prediction models constructed, which were mainly based on logistic regression. Sixteen of the included articles were cohort studies. Table 1 shows the basic information.

**Table 1:** Basic characteristics of the included studies.

No.	First author	Year of publication	Author country	Location	Study type	Source of patients	Disease	Diagnostic criteria for postthrombotic syndrome	Duration of follow-up	Number of cases of postthrombotic syndrome	Total number of cases	The number of postthrombotic syndrome cases in the training set	Total number of cases in the training set	Generation of the validation set	The number of postthrombotic syndrome cases in the validation set	Total number of cases in the validation set	Variable screening/feature selection method
1	Zhaoyu Wu (9)	2022	China	Asia	Cohort study	Single center	DVT	Villalta score ≥ 5	6 m	126	300	99	240	Random sampling	27	60	Logistic regression + decision tree
2	Wik HS (10)	2012	Norway	Europe	Case-control	Multi-center	Pregnancy-related venous thrombosis	Villalta score ≥ 5		156	623						Bivariate logistic regression analysis
3	Jiantao Zhang (11)	2022	China	Asia	Prospective cohort study	Single center	DVT	Villalta score ≥ 5	24 m	116	808	76	540	Prospective	40	268	The least absolute shrinkage and selection operator (LASSO) with 10-fold cross-validation
4	Tao Yu (12)	2022	China	Asia	Cohort study	Single center	DVT	Villalta score ≥ 5	24 m	365	672	327	555	Cross-validation	38	117	Logistic regression + decision tree
5	Félix Rinfret (13)	2022	Canada	North America	Cohort study	Multi-center	DVT	Villalta score ≥ 5	24 m	328	691						Logistic regression + univariate + multivariate
6	L. W. T ICK (14)	2010	Netherlands	Europe	Prospective cohort study	Single center	DVT	CEAP score ≥ 3	24 m	56	111						Multivariate logistic regression model
7	T. YAMAKI (15)	2007	Japan	Asia	Prospective cohort study	Single center	DVT	CEAP score ≥ 3	72 m	35	131						Multivariate logistic regression analysis
8	RHW Strijkers (16)	2015	Netherlands/Belgium	Europe	Cohort study	Multi-center	DVT	Villalta score ≥ 5/LET score(LETⅡ)		180	309						Multivariate logistic regression model
9	Farieda Ariyanti (17)	2023	Indonesia	Asia	Retrospective cohort study	Single center	DVT	Villalta score ≥ 5		49	91						Multivariate logistic regression model
10	Hao Huang (18)	2018	China	Asia	Cohort study	Single center	DVT	Villalta score ≥ 5/APTSD score (>7.0)	24 m	96	291	51	156	Random sampling	45	135	Independent factor (univariate)
11	Blake McLeod (19)	2022	Australia	Oceania	Prospective cohort study	Single center	DVT	Villalta score ≥ 5	36 m	62	190						Multivariate logistic regression model
12	Marie Méan (20)	2018	Switzerland	Europe	Prospective cohort study	Multi-center	DVT	Villalta score ≥ 5	24 m	161	276						Logistic regression analysis
13	Peng Qiu (21)	2021	China	Asia	Case-control (retrospective)	Single center	DVT	Villalta score ≥ 5	6 m	126	300	84	210	Random sampling	42	90	Univariate [select significant variables (*P* < 0.05) in single factor analysis to establish a multivariate logistic regression model]
14	A. Rabinovich (22)	2018	Israel	Asia	Prospective cohort study	Multi-center	DVT	Villalta score ≥ 5/SOX-PTS score ≥ 4	24 m	90	762						Multivariate logistic regression analysis
15	Anat Rabinovich (23)	2020	Israel	Asia	Prospective cohort study	Multi-center	DVT	Villalta score ≥ 5/SOX-PTS score ≥ 4	24 m	328	691	157	336	Random sampling	171	355	Multivariate logistic regression analysis
16	Nadeem A Siddiqui (24)	2016	Pakistan	Asia	Case-control	Single center	DVT	Villalta score ≥ 5		49	125						Multivariate
17	Elham E. Amin (25)	2018	Netherlands	Europe	Prospective cohort study	Multi-center	DVT	Villalta score ≥ 5	24 m	712	1558	236	451	Random sampling	476	1107	Multivariate logistic regression analysis
18	Sandra Mrozinska (26)	2018	Poland	Europe	Prospective cohort study	Single center	DVT	Villalta score ≥ 5	24 m	83	309						Multivariate logistic regression model/LOWESS
19	Maciej Wiktor Polak (27)	2019	Poland	Europe	Prospective cohort study	Single center	DVT	Villalta score ≥ 5	53 m	57	186						Univariate + multivariate Cox proportional hazards analysis to identify independent predictors of prognosis
20	Marit Engeseth (28)	2021	Norway	Europe	Case-control	Single center	DVT	Villalta score ≥ 5		44	88						Multivariate logistic regression model

### 3.3. Assessment of study quality

Newcastle-Ottawa Scale (NOS) was used to evaluate the quality of the included articles. There were six 9-point articles, seven 8-point articles, six 7-point articles, and one 6-point article. In the cohort studies, scores were not obtained mainly in the three areas of representativeness of the exposure cohort, assessment of outcome events, and completeness of follow-up. In case-control studies, there were no scores for control selection and non-response rates. The specific assessment results are provided in Table 2.

**Table 2:** Results of quality evaluation of the included literature by NOS scale.

No	Author	Year	v1	v2	v3	v4	v5	v6	v7	v8	v9	Total
1	Zhaoyu Wu	2022	0	1	1	1	1	1	1	0	1	7
2	Wik HS	2012	1	1	1	1	1	1	1	1	0	8
3	Jiantao Zhang	2022	1	1	1	1	1	1	0	1	1	8
4	Tao Yu	2022	1	0	1	1	1	1	1	1	1	8
5	Félix Rinfret	2022	1	1	1	1	1	1	1	1	0	8
6	L. W. T Ick	2010	1	1	0	1	1	1	1	1	1	8
7	T. Yamaki	2007	1	1	1	1	1	1	1	1	1	9
8	RHW Strijkers	2015	1	1	1	1	1	1	1	0	0	7
9	Farieda Ariyanti	2023	0	1	1	1	1	1	1	0	0	6
10	Hao Huang	2018	1	1	1	1	1	1	1	1	1	9
11	Blake McLeod	2022	1	1	1	1	1	1	1	1	1	9
12	Marie Méan	2018	1	1	1	1	1	1	1	1	1	9
13	Peng Qiu	2021	0	1	0	1	1	1	1	1	1	7
14	A. Rabinovich	2018	1	1	1	1	1	1	1	1	1	9
15	Anat Rabinovich	2020	1	1	0	1	1	1	0	1	1	7
16	Nadeem A Siddiqui	2016	1	1	0	1	1	1	1	1	1	8
17	Elham E. Amin	2018	1	0	1	1	1	1	0	1	1	7
18	Sandra Mrozinska	2018	1	1	1	1	1	1	1	1	1	9
19	Maciej Wiktor Polak	2019	1	1	1	1	1	1	0	1	1	8
20	Marit Engeseth	2021	1	1	0	1	1	1	1	1	0	7

### 3.4. Meta-analysis

#### 3.4.1. Incidence of post-thrombotic syndrome

##### 3.4.1.1. Synthesized results

**Figure 3:**
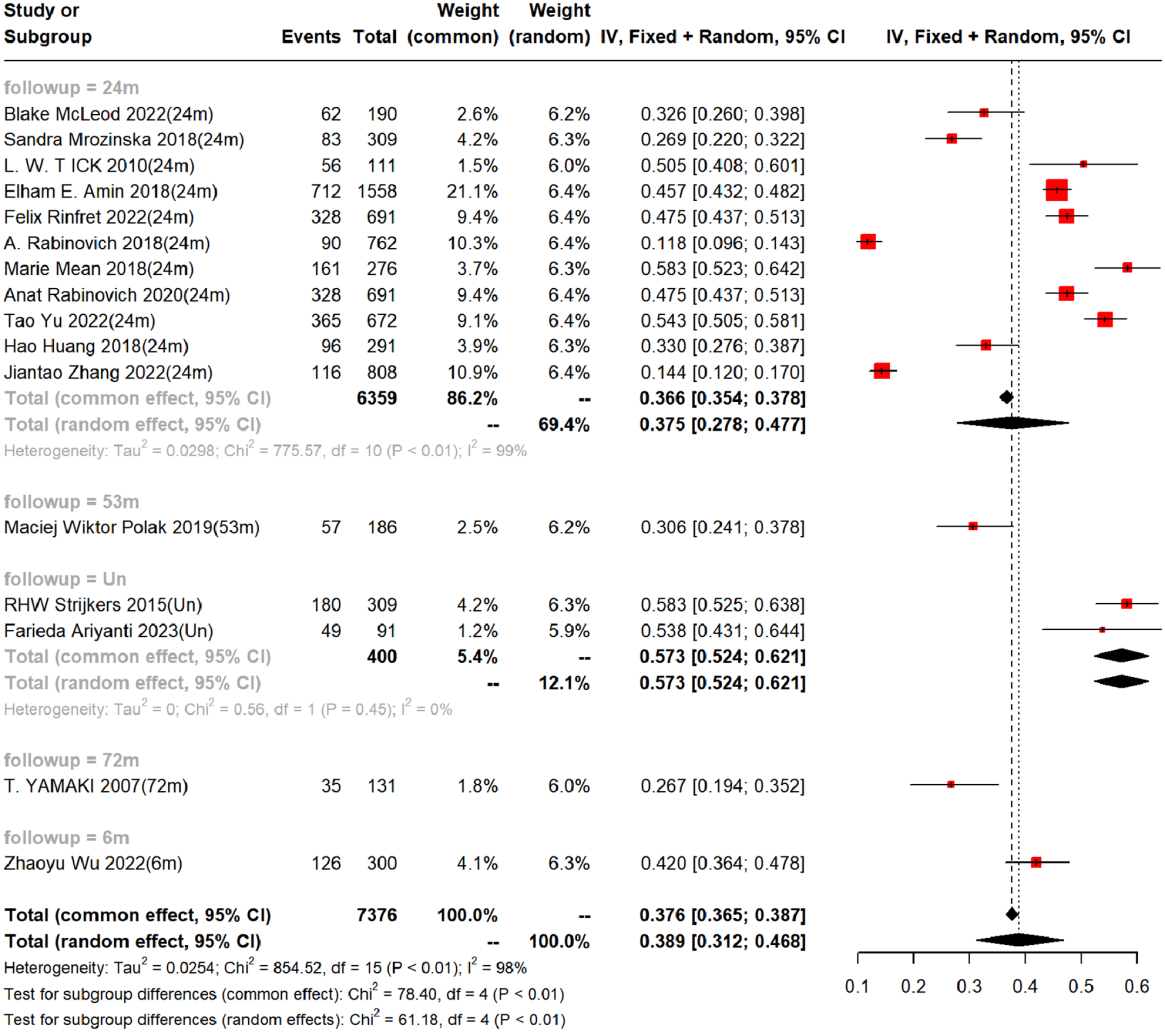
Incidence of PTS at different follow-up times. (Un- indicates no follow-up time described).

In the 16 cohort studies, the incidence of PTS was followed up for a maximum of 2 years, with a 2-year incidence of 37.5% (95%CI, 27.8–47.7%). In addition, there were differences in the incidence rates among countries within 2 years (Range: 26.86%−58.33%). Funnel plots showed the presence of publication bias between studies, while Begg's test and Egger's test revealed no publication bias between studies (Begg's test *P* = 0.928, Egger's test *P* = 0.096), as shown in Table 3, Figure 3 and Supplementary Figures S1–S2.

**Table 3:** Two-year incidence of PTS in different countries.

Country	*n*	In (95%CI)	*I*^2^ (%)
Australia	1	32.63% (26.13–39.48%)	NA
Poland	1	26.86% (22.06–31.95%)	NA
Netherlands	2	46.00% (43.60–48.40%)	0
Canada	1	47.47% (43.75–51.20%)	NA
Israel	2	26.92% (24.67–29.23%)	99.6
Switzerland	1	58.33% (52.46–64.09%)	NA
China	3	31.16% (29.02–33.34%)	99.3

#### 3.4.2. Independent predictors

These articles covered 51 predictors, with independent predictors primarily including old age, BMI, location of deep vein thrombosis, history of venous thrombosis, history of smoking, SOX-PTS score, varicose veins, and Villalta score. The OR values of these predictors were: old age OR = 1.840 (95% CI: 1.410–2.402; *I*^2 ^= 49%), obesity or overweight OR = 1.721 (95% CI: 1.245–2.378; *I*^2 ^= 56%), proximal deep vein thrombosis OR = 2.335 (95% CI: 1.855–2.938; *I*^2 ^= 0%), history of venous thrombosis OR = 3.593 (95% CI: 1.738–7.240; *I*^2 ^= 68%), history of smoking OR = 2.051 (95% CI: 1.305–3.224; *I*^2 ^= 7%), SOX -PTS score ≥4 OR = 4.806 (95% CI:2.921–7. 908; *I*^2 ^= 0%), varicose veins OR = 2.405 (95% CI:1.344–4.304; *I*^2 ^= 81%), and persistent increase in baseline Villalta score OR = 1.095 (95% CI:1.056–1.135; *I*^2 ^= 34%). We also found that gender, unprovoked DVT, and inadequate anticoagulation were not independent predictors (Table 4 and Supplementary Figures S3–S8).

**Table 4:** Meta-analysis results of early independent predictors of PTS.

Factors	Value	*n*	OR (95%CI)	*I* ^2^
Age				
	Old age	4	1.840 (1.410–2.402)	49.3
	Per 1 year	2	0.996 (0.930–1.067)	94.4
BMI				
	Obesity	7	1.721 (1.245–2.378)	56.4
	Per 1 kg/m^2^	3	1.174 (0.935–1.476)	88
Sex	Male	6	1.189 (0.780–1.812)	80.9
DVT localization	Proximal end	9	2.335 (1.855–2.938)	0
History of VTE	Yes	4	3.593 (1.738–7.240)	68.3
Smoking	Yes	2	2.051 (1.305–3.224)	6.5
SOX-PTS score				
	1 point	2	1.696 (1.191–2.415)	36.5
	2 points	2	2.085 (1.196–3.634)	50
	3 points	2	3.679 (2.431–5.569)	0
	≥4 points	2	4.806 (2.921–7.908)	0
Varicose veins	Yes	5	2.405 (1.344–4.304)	80.7
Unprovoked DVT	Yes	3	1.290 (0.734–2.226)	76.9
Villalta score category				
	Score 10–14	2	1.648 (1.230–2.208)	0
	Score >14	2	2.612 (1.837–3.715)	0
	Per 1 score	2	1.095 (1.056–1.135)	33.6
Insufficient anticoagulation	Yes	2	2.018 (0.812–5.019)	75.8

Moreover, among the remaining 39 predictors, we found that there was only one study for each predictor, and the results of these predictors need to be carefully interpreted. The detailed results are shown in Supplementary Material S2.

#### 3.4.3. Accuracy of the prediction model

**Figure 4:**
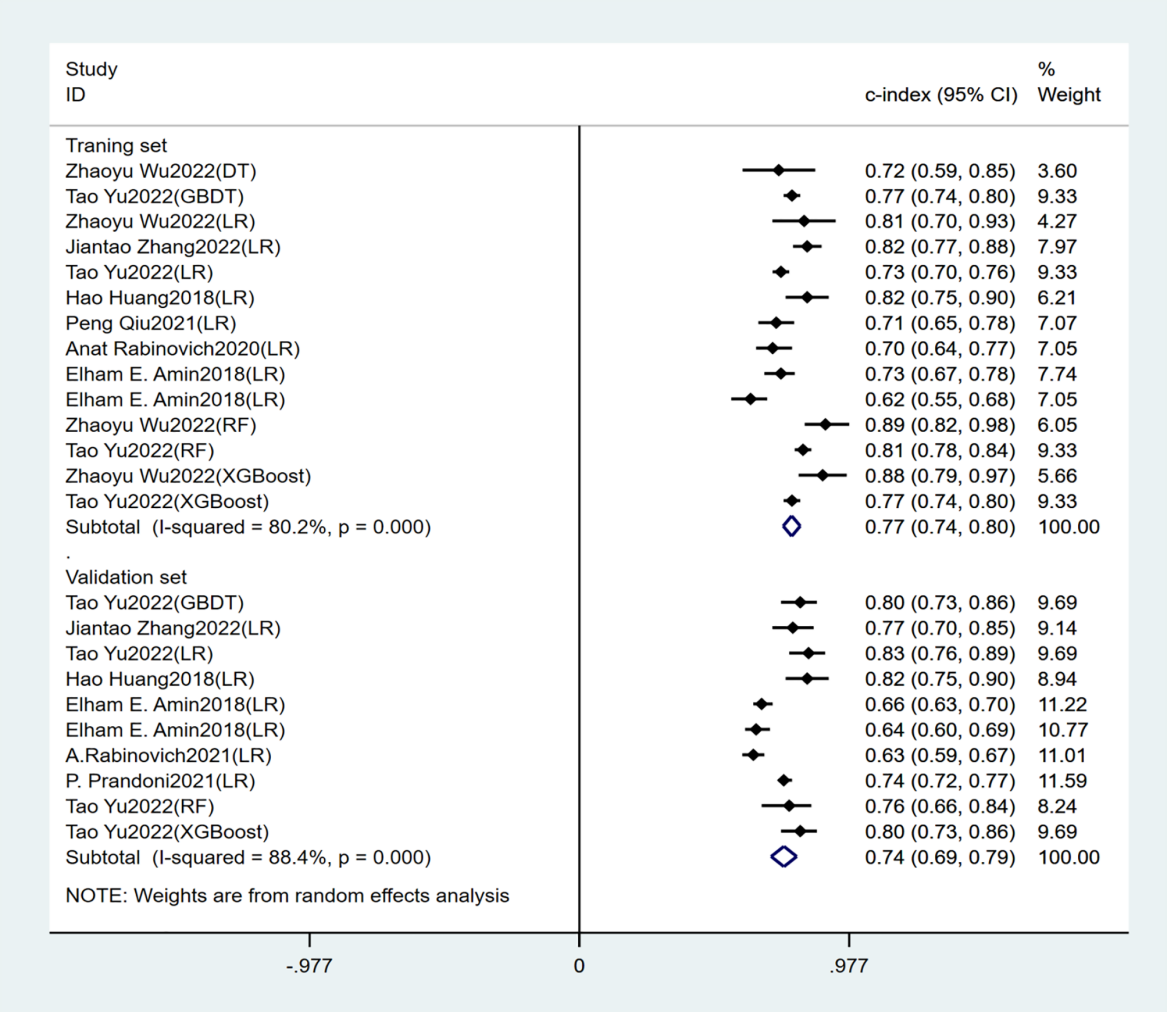
C-index of the training and validation sets of the early-constructed models.

**Figure 5:**
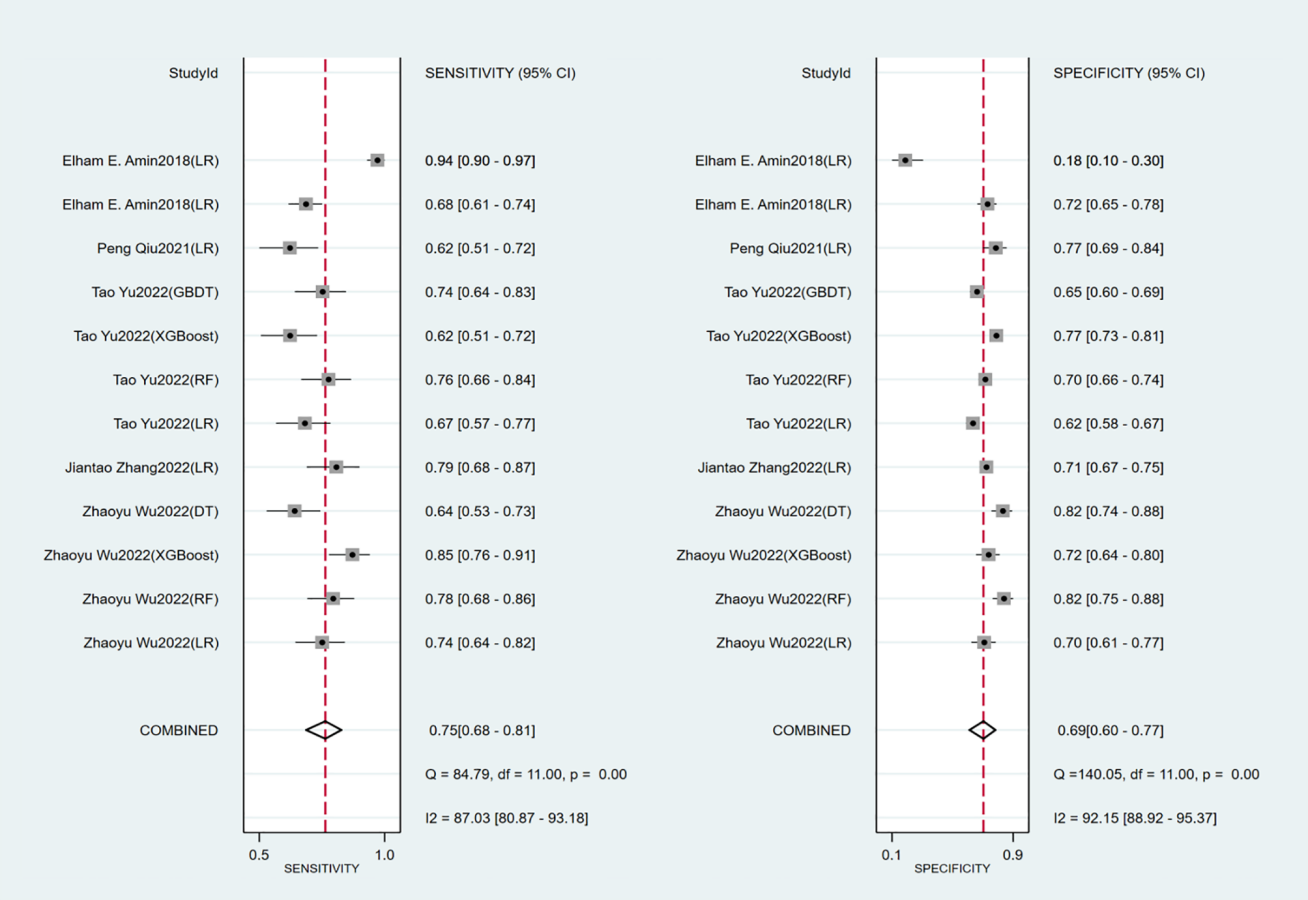
Sensitivity and specificity of the training set of the early-constructed models.

**Figure 6:**
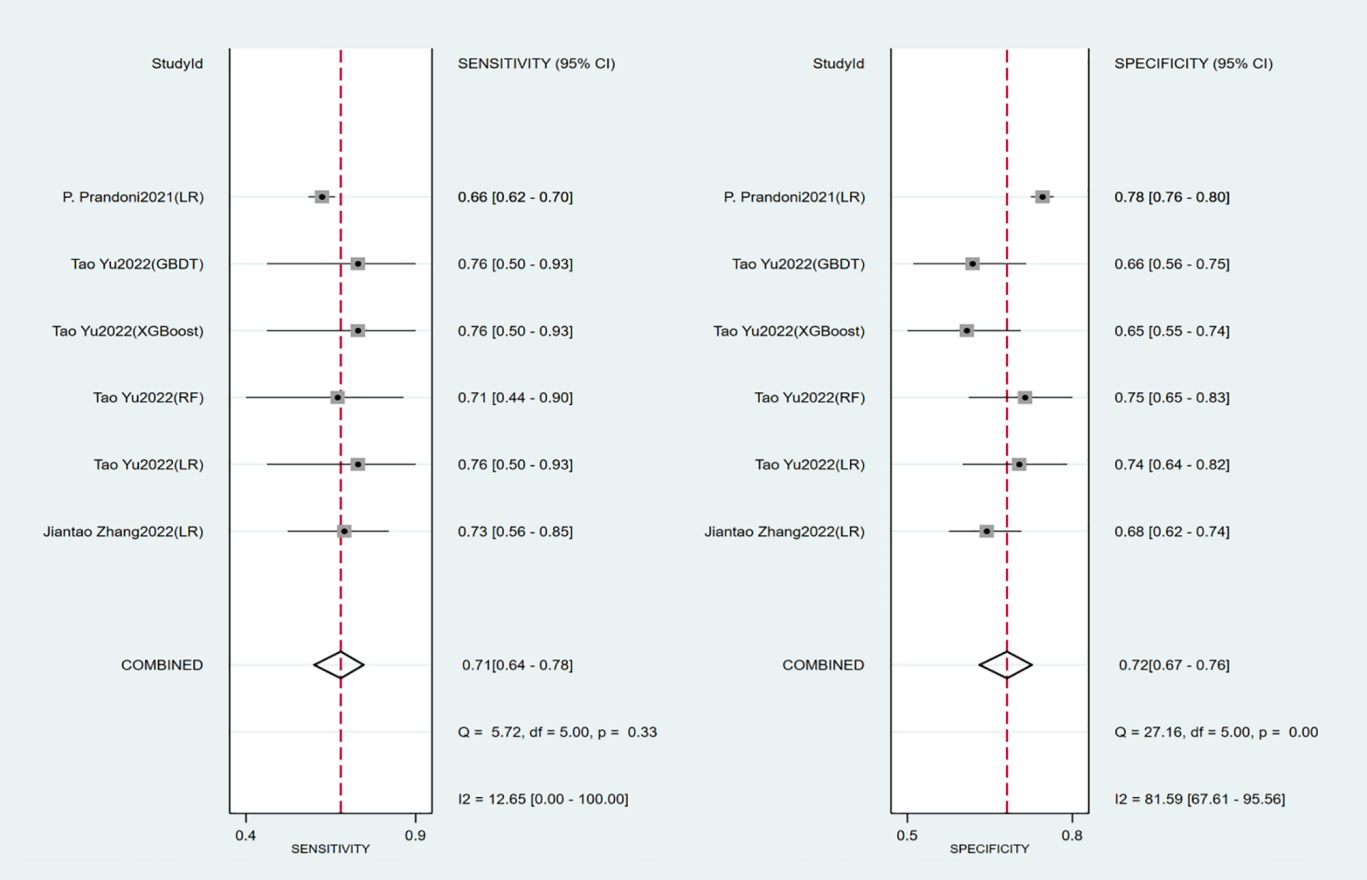
Sensitivity and specificity of the validation set of the early-constructed models.

Seven studies constructed risk prediction models. Most of them were nomograms constructed by logistic regression, covering a few other models such as decision trees, random forest, XGBoost, and GBDT. In the training set (Figures 4, 5), the c-index of the prediction models was 0.77 (95%CI: 0.74–0.80), the sensitivity was 0.75 (95%CI: 0.68–0.81) and the specificity was 0.69 (95%CI:0.60–0.77). In the validation set (Figures 4, 6), the c-index, sensitivity and specificity of the prediction models were 0.74 (95%CI: 0.69–0.79), 0.71 (95%CI: 0.64–0.78) and 0.72 (95% CI: 0.67–0.76), respectively. The detailed data are provided in Supplementary Tables S1, S2.

## 4. Discussion

### 4.1. Summary of the main findings

Our study showed that the follow-up of PTS was mainly within 3 years, with a focus on 2-year incidence. The incidence of PTS in our study was 37.5% (95% CI: 27.8–47.7%). We also found huge differences in incidence rates among countries, which may be related to the level of medical treatment and local concern, and We should increase awareness of clinical prevention and control of PTS.

Independent predictors in our systematic review included old age, BMI, DVT location, history of venous thrombosis, smoking history, SOX-PTS score, varicose veins, and baseline Villalta score. The prediction models constructed based on these predictors had good accuracy, with a c-index of 0.77 (95% CI: 0.74–0.80), sensitivity of 0.75 (95% CI: 0.68–0.81), and specificity of 0.69 (95% CI: 0.60–0.77) in the training set. In the validation set, the c-index, sensitivity and specificity of the prediction models were 0.74 (95% CI: 0.69–0.79), 0.71 (95% CI: 0.64–0.78) and 0.72 (95% CI: 0.67–0.76) respectively.

### 4.2. Comparison with previous studies

Most reviews on PTS focus on summarizing relevant studies on PTS (e.g., long-term therapy (29), anticoagulant selection (30), catheter therapy (31, 32), etc.), describing and explaining the main results, findings and trends of the studies, and drawing conclusions from them. The PTS reviews describe the pathophysiology, risk factors, diagnosis, prevention, and treatment of PTS to improve our understanding of the disease and guide treatment.

As found in the review, two articles [(Kearon et al., 2020 (29) and Kruger et al., 2019 (30)] proposed male as an risk factor of PTS, which is contradictory with our findings. In this study, male OR = 1.189 (95%CI; 0.780–1.812) was not a risk factor of PTS. Meanwhile, Iding AFJ et al., 2023 (33) noted in their study that estrogen use in women reduces the incidence of PTS, so further research is needed to determine whether gender can be used as an independent predictor. Unprovoked DVT is DVT occurring in the absence of a transient risk factor. And the review of Kruger et al. (2019) described unprovoked DVT as an independent predictor, which differs from our findings, as in this paper unprovoked DVT OR = 1.291 (95% CI; 0.742–2.246) could not be regarded as an independent predictor. However, it has been suggested in one article (33) that Unprovoked DVT is closely related to the patient's cardiovascular disease. Therefore, it can be inferred that whether Unprovoked DVT can be an independent predictor is inextricably linked to the status of its study patients.The above may be due to the heterogeneity of the DVT patient population in the studies that included the above predictors and the diversity of clinical manifestations of PTS, leading to different results for independent predictors (34). Based on the present study and several previous studies, the main independent predictors of PTS were old age, obesity, proximal DVT, venous thromboembolism history, and baseline Villalta score. Therefore, the remaining controversial predictors require more research to explore the independent predictors of PTS.

Galanaud et al. 2018 (35), Kearon et al. 2020, INOVICH et al. 2017 (36), and Makedonov et al. 2020 (37) all noted in their studies that the prevalence of PTS was about 20%–50%, of which 5%–10% of patients would develop severe PTS. Kruger et al. 2019 (30) pointed out in a study that the incidence of PTS was 40%. In a meta-analysis (38) on the combined risk of PTS, mainly the risk of PTS caused by distal venous thrombosis, it was estimated that 20% of patients with venous thrombosis would develop PTS, among which 20% would develop severe PTS. The main background of this study is proximal venous thrombosis, the prevalence of which is 37.5% in this paper, probably due to differences in study countries, study populations, tools used to assess PTS, and the time interval between DVT and PTS assessment, making the prevalence of PTS reported in the studies highly variable.

Meanwhile, PTS is mentioned in several studies as significantly reducing the quality of life and being expensive, so early prediction and intervention for PTS is an important direction for treatment (30, 36, 37, 39). Development of risk predictors to predict the risk of PTS at the time of diagnosis of DVT is also ongoing to help guide the long-term treatment of patients with DVT (36). The purpose of this systematic meta-analysis was to comprehensively analyze data on incidence, predictors, and the accuracy and feasibility of predictive models from multiple studies so as to evaluate the predictive value of predictors and machine learning-based predictive models. This study allows us to further explore the causes of morbidity fluctuations, analyze the value of independent predictors and provide a reference for the development of more accurate and reliable machine prediction models, and the conclusions obtained can contribute to future relevant medical decision-making and disease prevention and treatment.

### 4.3. Advantages and limitations of the study

In this review, a consensus was reached by two independent reviewers at the literature selection stage to reduce the risk of selection bias. Further, this study is the first to focus on the incidence and predictors of PTS from the perspective of a systematic review. A comprehensive analysis of multiple independent predictors eliminates bias and noise in the study, thus estimating the overall effects and confidence intervals more accurately. Meanwhile, by examining the heterogeneity and consistency in different studies, the accuracy and feasibility of the early prediction models of PTS were further revealed, and the reliability of the conclusions was improved.

There are also some limitations in this paper. The number of cases in our included cohort studies was small, which may be due to the insufficient attention paid to venous thrombosis in clinical practice or some countries. Also, some of the predictors were included in a small number of literature reports and need to be interpreted with caution. Among the included literature, a small part of them have built risk prediction models, and thus more large-sample studies are needed for verification.

## 5. Conclusions

As a complication that cannot be ignored in patients with venous thrombosis, PTS not only has a significant impact on the quality of life, but also causes important health and economic issues. In this case, risk prediction scoring based on early model construction is a feasible scheme, which enables more accurate prediction of diseases with the support of data, so that both physicians and patients can make timely and targeted treatment and preventive measures.

## Data Availability

The original contributions presented in the study are included in the article/**Supplementary Material**, further inquiries can be directed to the corresponding author.
