# Supplementary material for: A systematic evaluation and meta-analysis of early prediction of post-thrombotic syndrome
Source: Front Cardiovasc Med. 2023 Aug 24;10:1250480. doi: 10.3389/fcvm.2023.1250480 (PMC10484413; doi:10.3389/fcvm.2023.1250480)
Supplement: Supplementary file 1 [file Table1.docx]

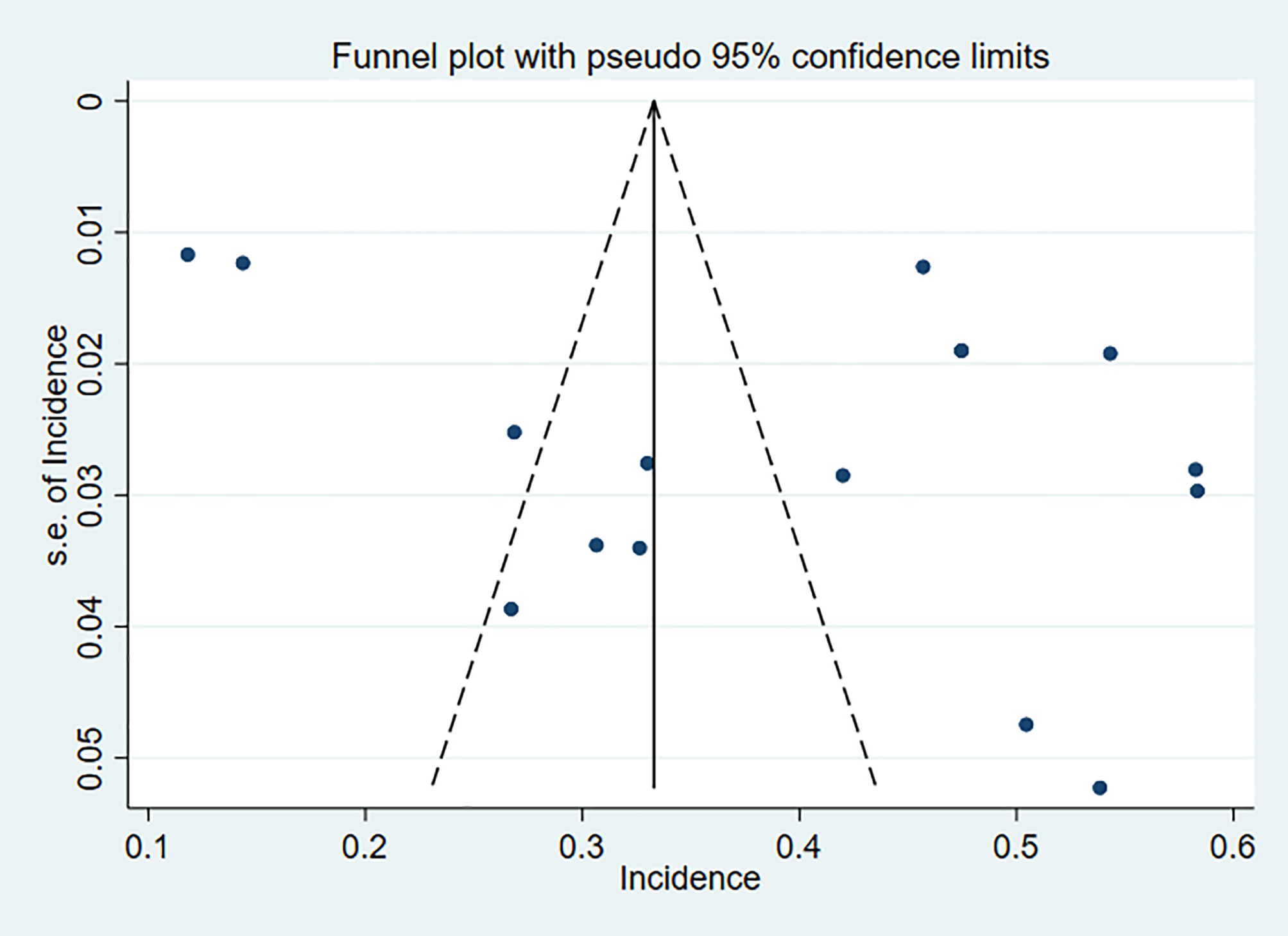


**Figure S1:** Funnel plot with pseudo 95% confidence limits


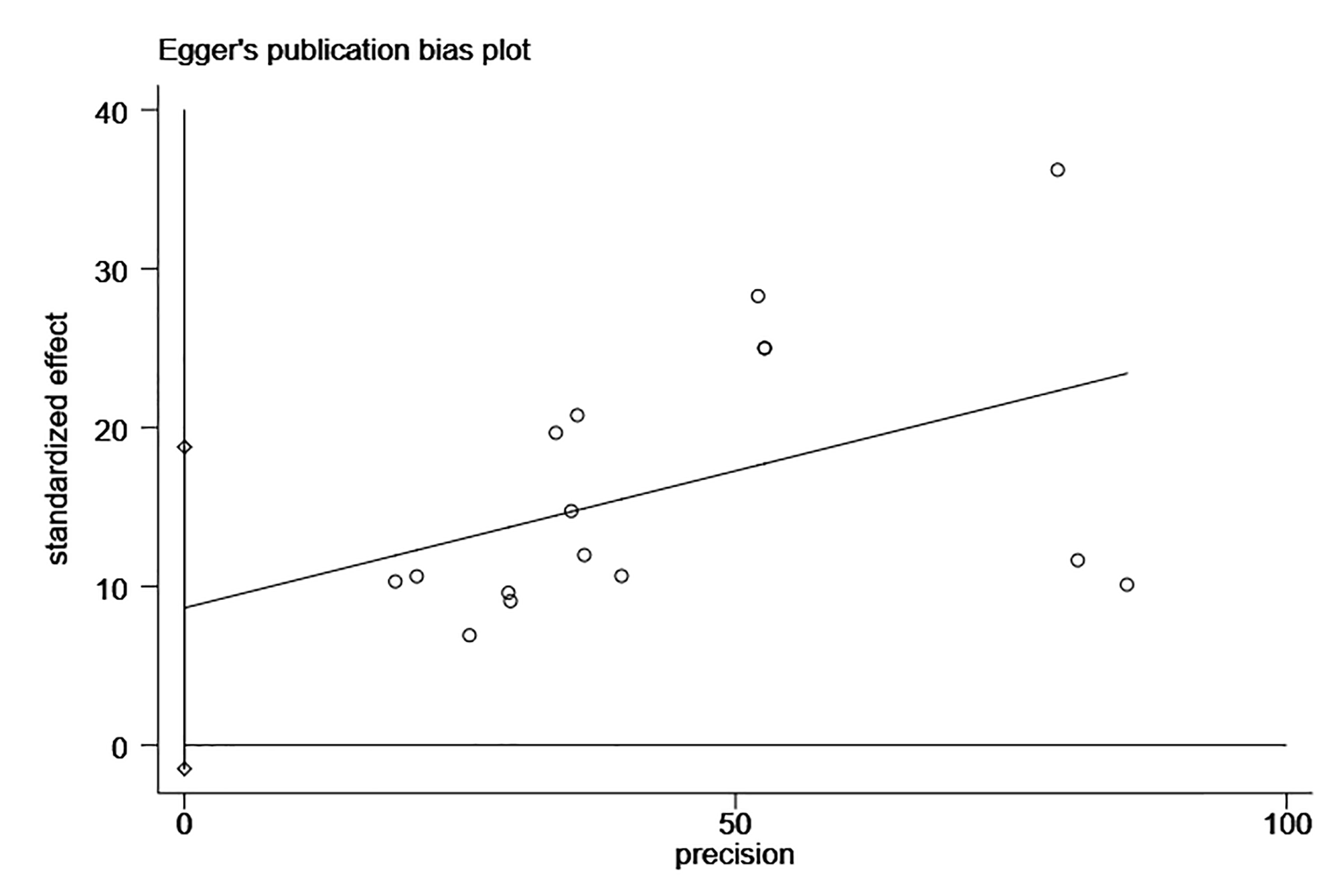


**Figure S2:** Egger’s publication bias plot


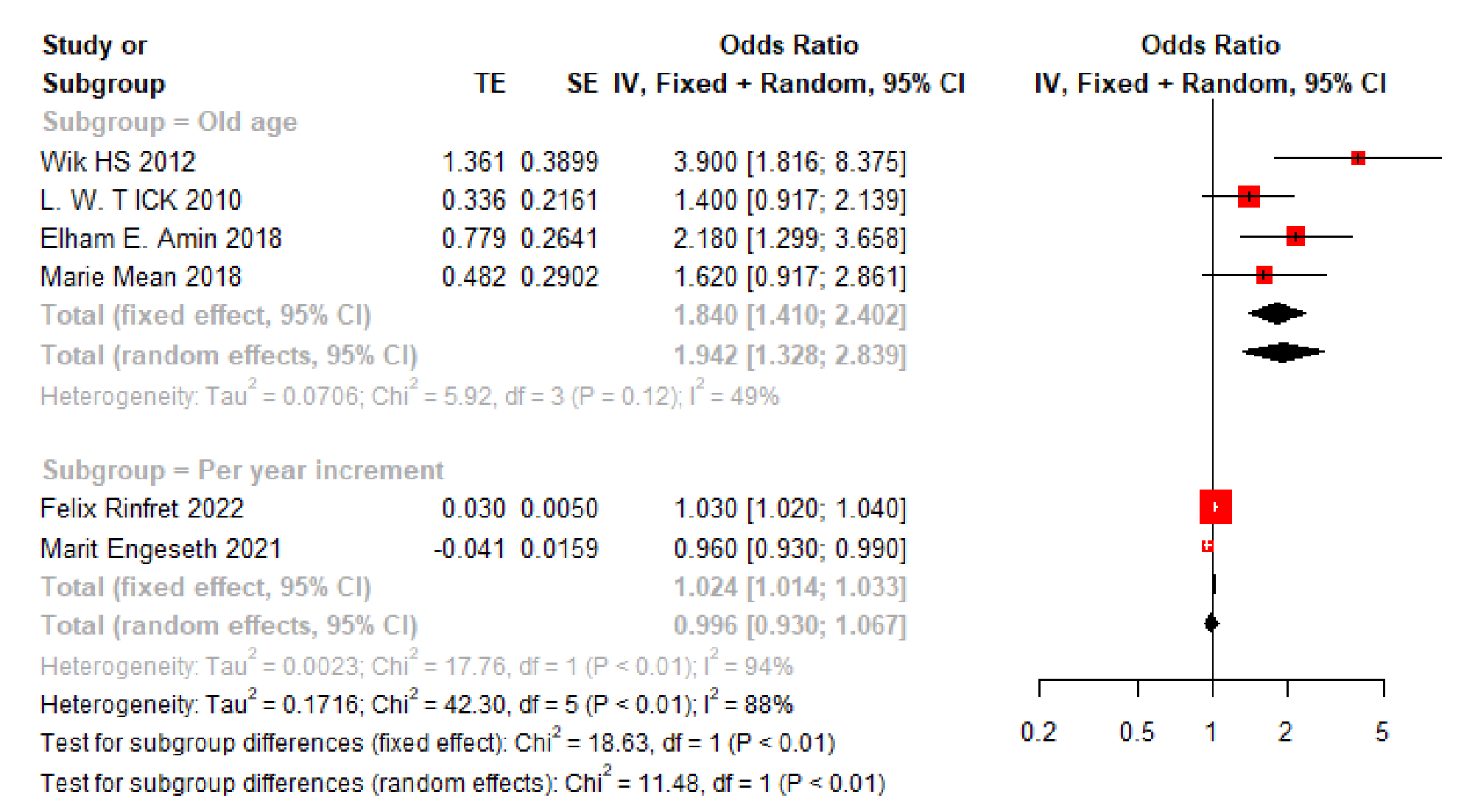


**Figure S3:** Forest plots of age


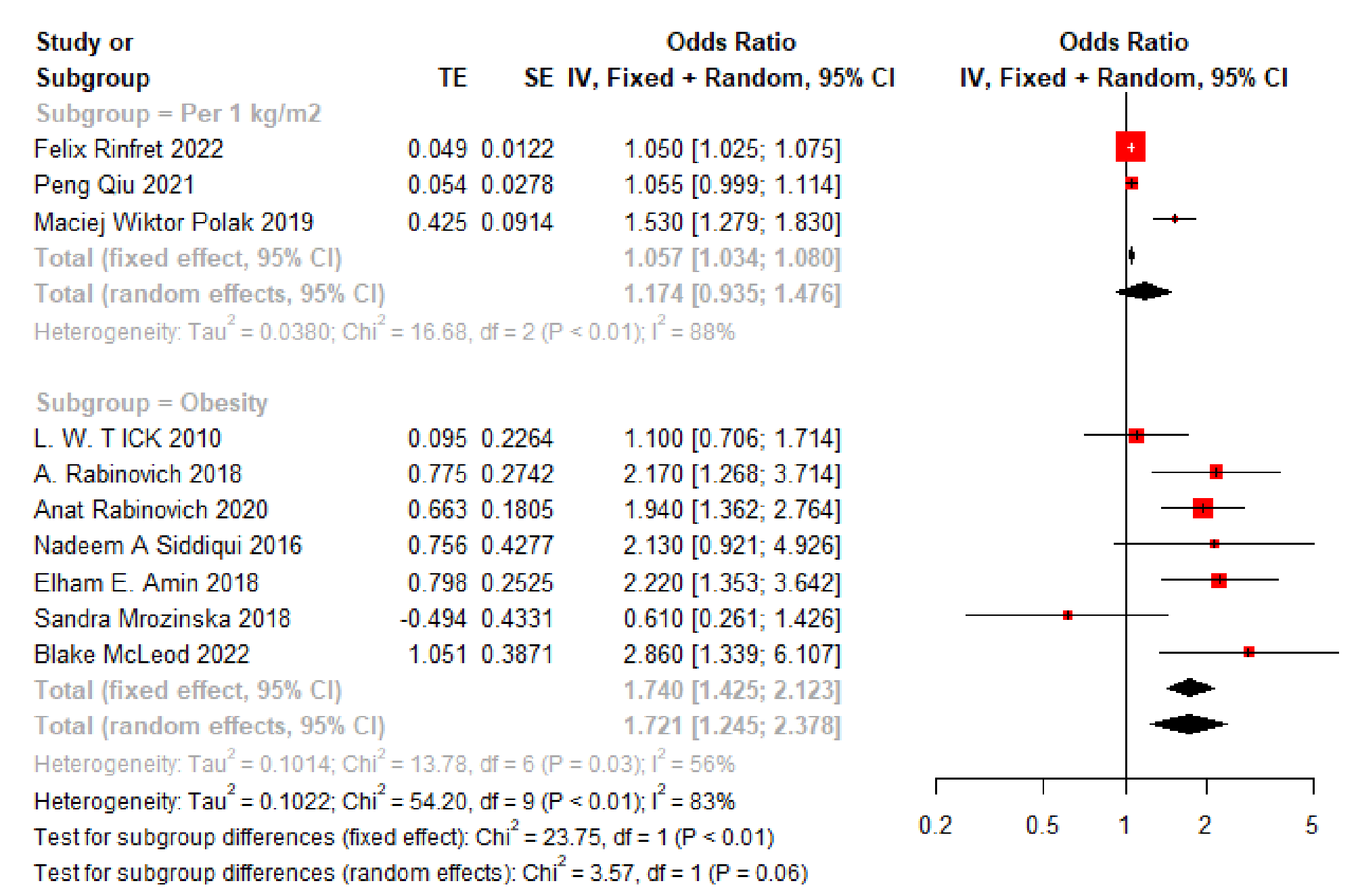


**Figure S4:** Forest plots of BMI


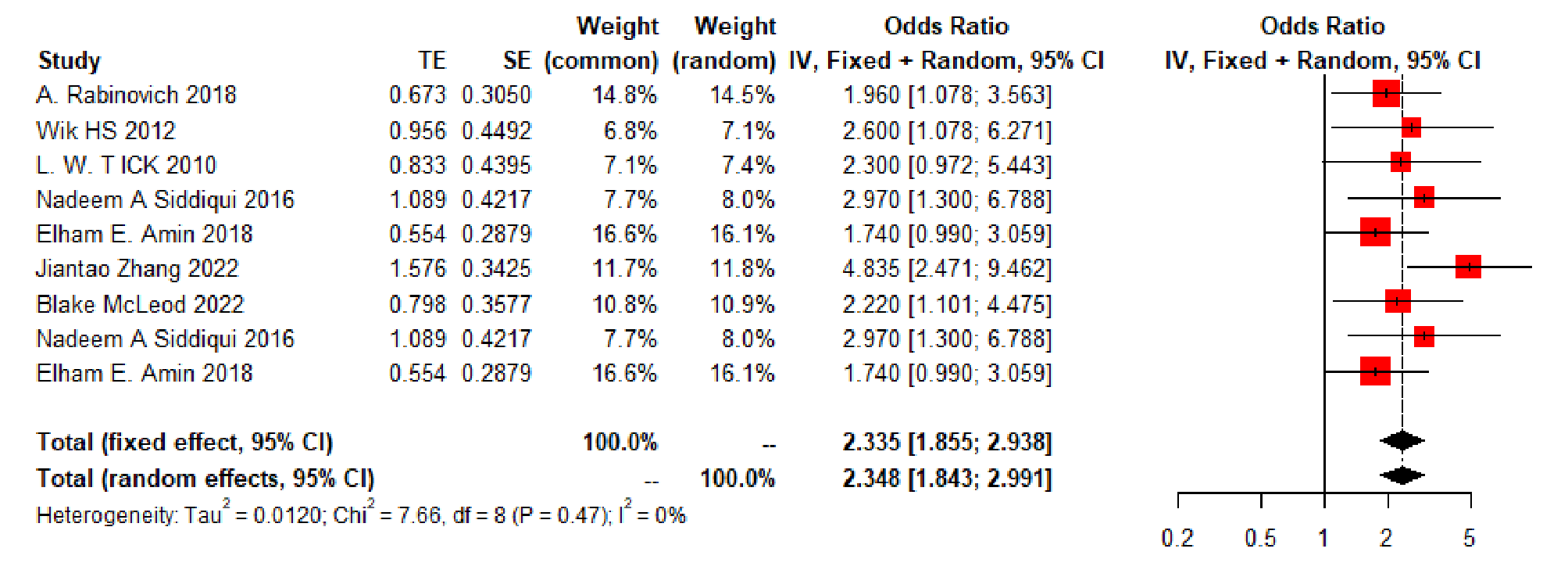


**Figure S5:** Forest plots of DVT localization


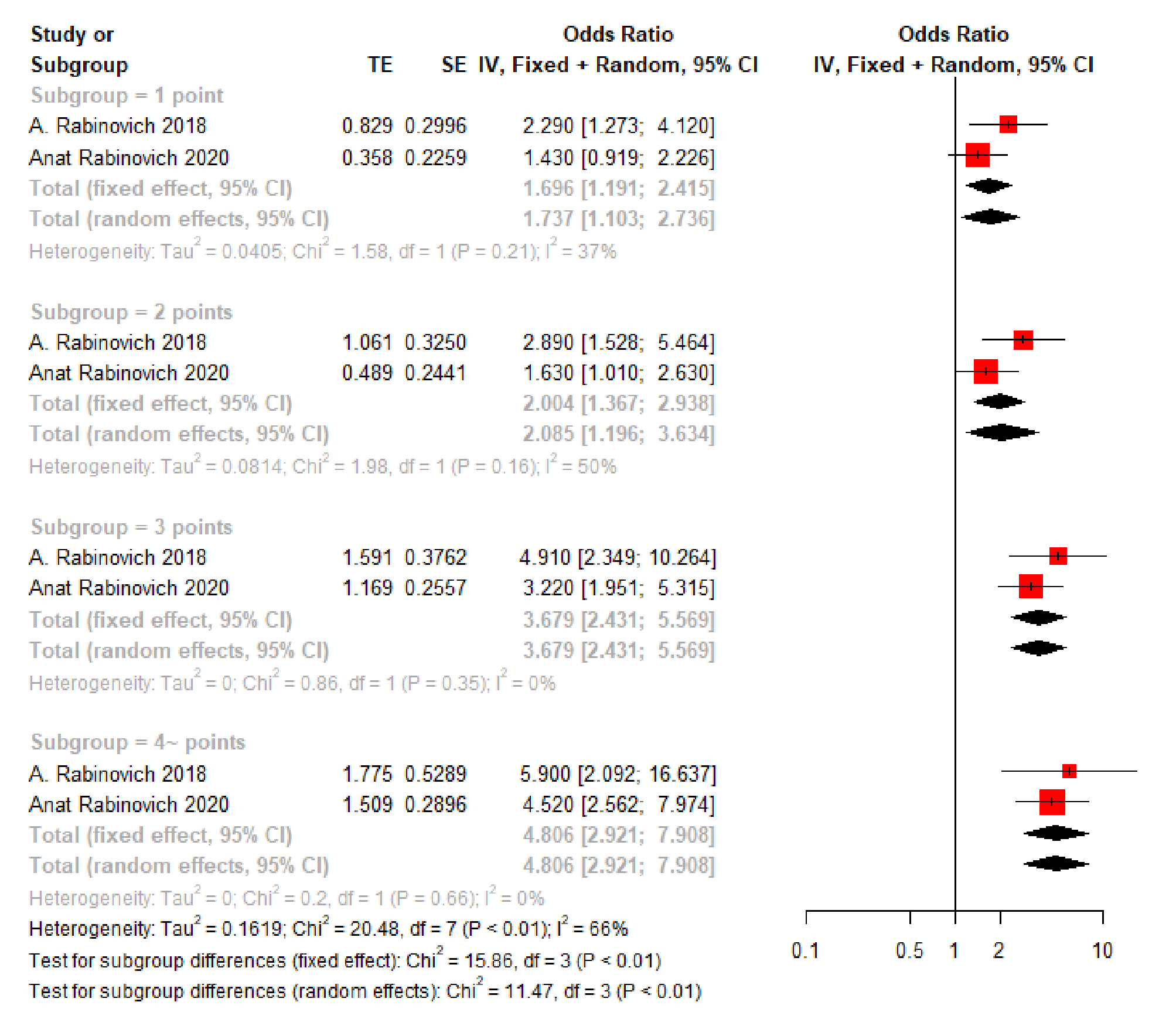


**Figure S6:** Forest plots of SOX-PTS Score


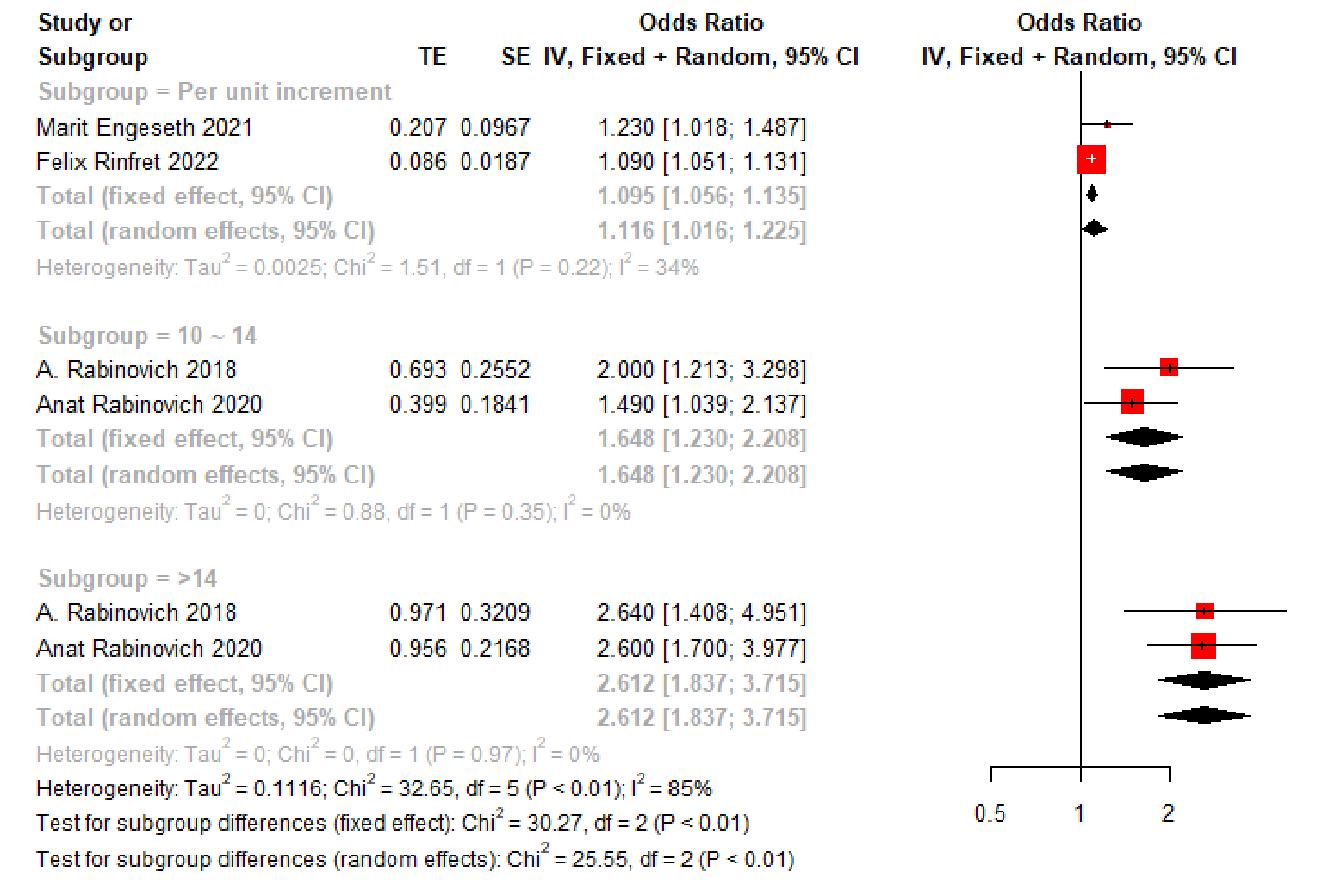


**Figure S7:** Forest plots of Villalta score category


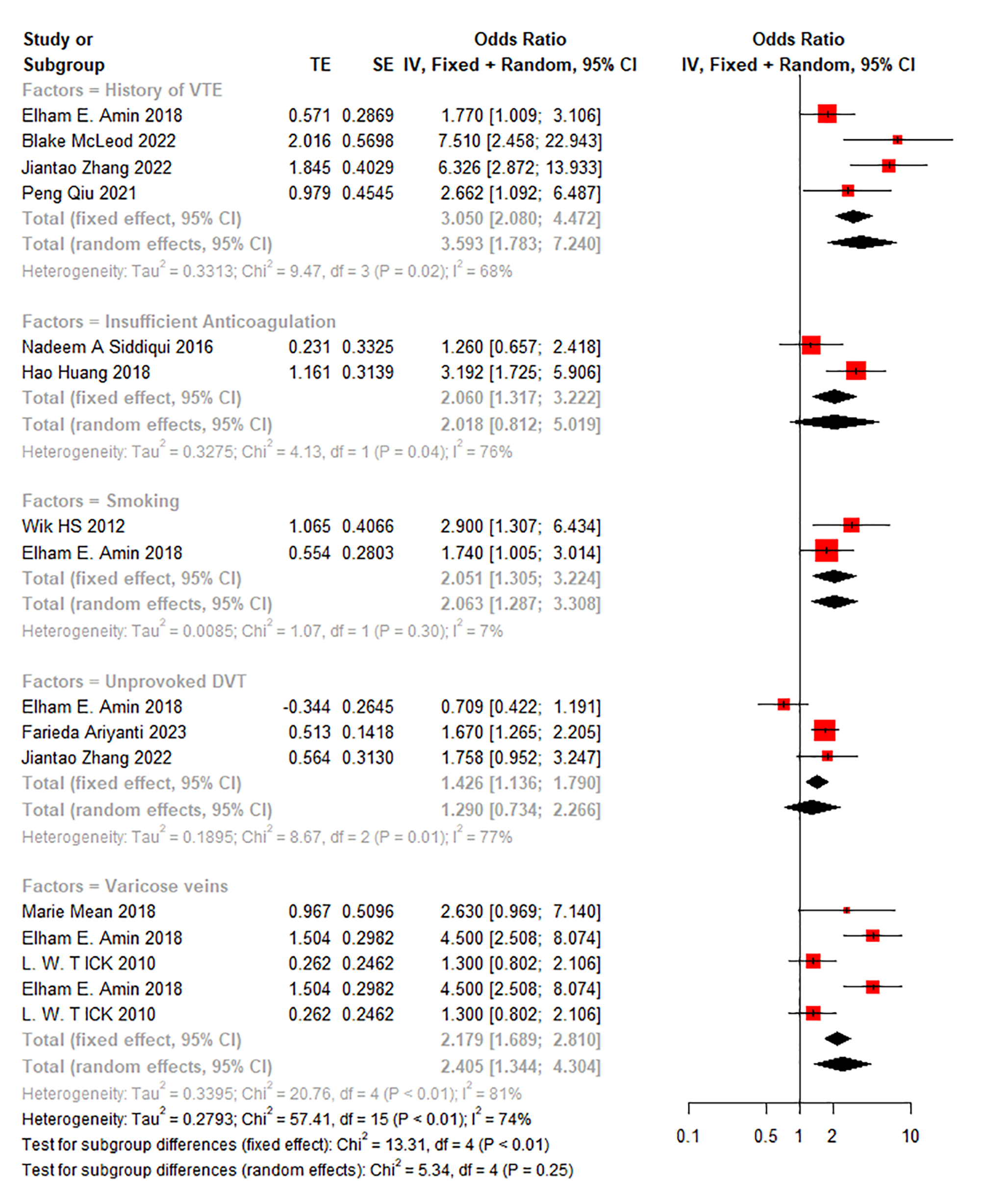


**Figure S8:** Forest plots of other main predictors
