# Supplementary material for: A systematic evaluation and meta-analysis of early prediction of post-thrombotic syndrome
Source: Front Cardiovasc Med. 2023 Aug 24;10:1250480. doi: 10.3389/fcvm.2023.1250480 (PMC10484413; doi:10.3389/fcvm.2023.1250480)
Supplement: Supplementary file 2 [file Table2.docx]

**Supplementary materials 1**

Literature search strategy

**1.Pubmed**

| Search number | Query | Results |
| --- | --- | --- |
| #1 | "Postthrombotic Syndrome"[Mesh] | 910 |
| #2 | (((((Postthrombotic Syndrome[Title/Abstract]) OR (Venous Stasis Syndrome[Title/Abstract])) OR (Post-thrombotic Syndrome[Title/Abstract])) OR (post thrombotic syndrome[Title/Abstract])) OR (postthrombophlebitic syndrome[Title/Abstract])) OR (postthrombosis syndrome[Title/Abstract]) | 2,212 |
| #3 | ("Postthrombotic Syndrome"[Mesh]) OR ((((((Postthrombotic Syndrome[Title/Abstract]) OR (Venous Stasis Syndrome[Title/Abstract])) OR (Post-thrombotic Syndrome[Title/Abstract])) OR (post thrombotic syndrome[Title/Abstract])) OR (postthrombophlebitic syndrome[Title/Abstract])) OR (postthrombosis syndrome[Title/Abstract])) | 2,434 |
| #4 | "Machine Learning"[Mesh] | 55,496 |
| #5 | (((((((((((((((((((((((((((Transfer Learning[Title/Abstract]) OR (Deep learning[Title/Abstract])) OR (Ensemble Learning[Title/Abstract])) OR (artificial intelligence[Title/Abstract])) OR (Prediction model[Title/Abstract])) OR (random forest[Title/Abstract])) OR (neural network[Title/Abstract])) OR (neural networks[Title/Abstract])) OR (Support vector machine[Title/Abstract])) OR (Gradient Boosting Machine[Title/Abstract])) OR (Nomogram[Title/Abstract])) OR (XGBoost[Title/Abstract])) OR (Adaboost[Title/Abstract])) OR (Decision tree[Title/Abstract])) OR (ResNet-50[Title/Abstract])) OR (ResNet[Title/Abstract])) OR (Radiomics[Title/Abstract])) OR (Radiomic[Title/Abstract])) OR (Naive Bayesian[Title/Abstract])) OR (Multilayer perceptron[Title/Abstract])) OR (Bayesian network[Title/Abstract])) OR (Risk Score[Title/Abstract])) OR (Risk model[Title/Abstract])) OR (c-statistic[Title/Abstract])) OR (Logistic[Title/Abstract])) OR (Risk factors[Title/Abstract])) OR (prediction factors[Title/Abstract])) OR (Predictors[Title/Abstract]) | 1,338,720 |
| #6 | ("Machine Learning"[Mesh]) OR ((((((((((((((((((((((((((((Transfer Learning[Title/Abstract]) OR (Deep learning[Title/Abstract])) OR (Ensemble Learning[Title/Abstract])) OR (artificial intelligence[Title/Abstract])) OR (Prediction model[Title/Abstract])) OR (random forest[Title/Abstract])) OR (neural network[Title/Abstract])) OR (neural networks[Title/Abstract])) OR (Support vector machine[Title/Abstract])) OR (Gradient Boosting Machine[Title/Abstract])) OR (Nomogram[Title/Abstract])) OR (XGBoost[Title/Abstract])) OR (Adaboost[Title/Abstract])) OR (Decision tree[Title/Abstract])) OR (ResNet-50[Title/Abstract])) OR (ResNet[Title/Abstract])) OR (Radiomics[Title/Abstract])) OR (Radiomic[Title/Abstract])) OR (Naive Bayesian[Title/Abstract])) OR (Multilayer perceptron[Title/Abstract])) OR (Bayesian network[Title/Abstract])) OR (Risk Score[Title/Abstract])) OR (Risk model[Title/Abstract])) OR (c-statistic[Title/Abstract])) OR (Logistic[Title/Abstract])) OR (Risk factors[Title/Abstract])) OR (prediction factors[Title/Abstract])) OR (Predictors[Title/Abstract])) | 1,355,539 |
| #7 | (("Postthrombotic Syndrome"[Mesh]) OR ((((((Postthrombotic Syndrome[Title/Abstract]) OR (Venous Stasis Syndrome[Title/Abstract])) OR (Post-thrombotic Syndrome[Title/Abstract])) OR (post thrombotic syndrome[Title/Abstract])) OR (postthrombophlebitic syndrome[Title/Abstract])) OR (postthrombosis syndrome[Title/Abstract]))) AND (("Machine Learning"[Mesh]) OR ((((((((((((((((((((((((((((Transfer Learning[Title/Abstract]) OR (Deep learning[Title/Abstract])) OR (Ensemble Learning[Title/Abstract])) OR (artificial intelligence[Title/Abstract])) OR (Prediction model[Title/Abstract])) OR (random forest[Title/Abstract])) OR (neural network[Title/Abstract])) OR (neural networks[Title/Abstract])) OR (Support vector machine[Title/Abstract])) OR (Gradient Boosting Machine[Title/Abstract])) OR (Nomogram[Title/Abstract])) OR (XGBoost[Title/Abstract])) OR (Adaboost[Title/Abstract])) OR (Decision tree[Title/Abstract])) OR (ResNet-50[Title/Abstract])) OR (ResNet[Title/Abstract])) OR (Radiomics[Title/Abstract])) OR (Radiomic[Title/Abstract])) OR (Naive Bayesian[Title/Abstract])) OR (Multilayer perceptron[Title/Abstract])) OR (Bayesian network[Title/Abstract])) OR (Risk Score[Title/Abstract])) OR (Risk model[Title/Abstract])) OR (c-statistic[Title/Abstract])) OR (Logistic[Title/Abstract])) OR (Risk factors[Title/Abstract])) OR (prediction factors[Title/Abstract])) OR (Predictors[Title/Abstract]))) | 366 |

**2.Cochrane**

| Search number | Query | Results |
| --- | --- | --- |
| #1 | MeSH descriptor: [Postthrombotic Syndrome] explode all trees | 135 |
| #2 | (Postthrombotic Syndrome):ti,ab,kw OR (Venous Stasis Syndrome):ti,ab,kw OR (Post-thrombotic Syndrome):ti,ab,kw OR (post thrombotic syndrome):ti,ab,kw OR (postthrombophlebitic syndrome):ti,ab,kw | 534 |
| #3 | (postthrombosis syndrome):ti,ab,kw | 178 |
| #4 | #1 or #2 or #3 | 547 |
| #5 | MeSH descriptor: [Machine Learning] explode all trees | 866 |
| #6 | (machine learning):ti,ab,kw OR (Transfer Learning):ti,ab,kw OR (Deep learning):ti,ab,kw OR (Ensemble Learning):ti,ab,kw OR (artificial intelligence):ti,ab,kw | 5,949 |
| #7 | (Prediction model):ti,ab,kw OR (random forest):ti,ab,kw OR (neural network):ti,ab,kw OR (neural networks):ti,ab,kw OR (Support vector machine):ti,ab,kw | 8,978 |
| #8 | (Gradient Boosting Machine):ti,ab,kw OR (Nomogram):ti,ab,kw OR (XGBoost):ti,ab,kw OR (Adaboost):ti,ab,kw OR (Decision tree):ti,ab,kw | 2,436 |
| #9 | (ResNet-50):ti,ab,kw OR (ResNet):ti,ab,kw OR (Radiomics):ti,ab,kw OR (Radiomic):ti,ab,kw OR (Naive Bayesian):ti,ab,kw | 690 |
| #10 | (Multilayer perceptron):ti,ab,kw OR (Bayesian network):ti,ab,kw OR (Risk Score):ti,ab,kw OR (Risk model):ti,ab,kw OR (c-statistic):ti,ab,kw | 60,662 |
| #11 | (Logistic):ti,ab,kw OR (Risk factors):ti,ab,kw OR (prediction factors):ti,ab,kw OR (Predictors):ti,ab,kw | 127,694 |
| #12 | #5 or #6 or #7 or #8 or #9 or #10 or #11 | 173,143 |
| #13 | #4 and #12 | 141 |

**3.Embase**

| Search number | Query | Results |
| --- | --- | --- |
| #1 | 'postthrombosis syndrome'/exp | 3,543 |
| #2 | 'postthrombotic syndrome':ab,ti OR 'venous stasis syndrome':ab,ti OR 'post-thrombotic syndrome':ab,ti OR 'post thrombotic syndrome':ab,ti OR 'postthrombophlebitic syndrome':ab,ti OR 'postthrombosis syndrome':ab,ti | 3,475 |
| #3 | #1 OR #2 | 4,569 |
| #4 | 'machine learning'/exp | 377,067 |
| #5 | 'machine learning':ab,ti OR 'transfer learning':ab,ti OR 'deep learning':ab,ti OR 'ensemble learning':ab,ti OR 'artificial intelligence':ab,ti OR 'prediction model':ab,ti OR 'random forest':ab,ti OR 'neural network':ab,ti OR 'neural networks':ab,ti OR 'support vector machine':ab,ti OR 'gradient boosting machine':ab,ti OR nomogram:ab,ti OR xgboost:ab,ti OR adaboost:ab,ti OR 'decision tree':ab,ti OR 'resnet 50':ab,ti OR resnet:ab,ti OR radiomics:ab,ti OR radiomic:ab,ti OR 'naive bayesian':ab,ti OR 'multilayer perceptron':ab,ti OR 'bayesian network':ab,ti OR 'risk score':ab,ti OR 'c statistic':ab,ti OR logistic:ab,ti OR 'risk factors':ab,ti OR 'prediction factors':ab,ti OR predictors:ab,ti | 1,911,104 |
| #6 | #4 OR #5 | 2,103,454 |
| #7 | #3 AND #6 | 723 |

**4.Web of science**

| Search number | Query | Results |
| --- | --- | --- |
| #1 | Postthrombotic Syndrome (Topic) OR Venous Stasis Syndrome (Topic) OR Post-thrombotic Syndrome ((Topic)) OR post thrombotic syndrome (Topic) OR postthrombophlebitic syndrome (Topic) OR postthrombosis syndrome (Topic) | 3,524 |
| #2 | machine learning (Topic) OR Transfer Learning (Topic) OR Deep learning (Topic) OR Ensemble Learning (Topic) OR artificial intelligence (Topic) OR Prediction model (Topic) OR random forest (Topic) OR neural network (Topic) OR neural networks (Topic) OR Support vector machine (Topic) OR Gradient Boosting Machine (Topic) OR Nomogram (Topic) OR XGBoost (Topic) OR Adaboost (Topic) OR Decision tree (Topic) OR ResNet-50 (Topic) OR ResNet (Topic) OR Radiomics (Topic) OR Radiomic (Topic) OR Naive Bayesian (Topic) OR Multilayer perceptron (Topic) OR Bayesian network (Topic) OR Risk Score (Topic) OR Risk model (Topic) OR c-statistic (Topic) OR Logistic (Topic) OR Risk factors (Topic) OR prediction factors (Topic) OR Predictors (Topic) | 4,831,888 |
| #3 | #1 AND #2 | 1,145 |

**Supplementary materials 2:** Odds ratio of the remaining 39 predictors of PTS

| Factors | Value | OR(95%CI) |
| --- | --- | --- |
| Multi-level thrombosis | yes | 1.74(0.97-3.12) |
| Active cancer | yes | 3.006(1.404-6.435) |
| Treatment allocation | pharmacomechanical catheter-direc ted thrombolysis | 0.87(0.61-1.22) |
| Adiponectin | ＞1 µg/mL | 0.42(0.31-0.56) |
| Chronic deep venous thrombosis | yes | 2.463(1.397-4.393) |
| Chronic kidney disease | yes | 9.916(2.238-43.937) |
| Chronic venous insufficiency | yes | 7.464(3.568-15.616) |
| Employment status(employed ≥35 h/week Ref) |  |  |
|  | Employed <35 hours per week | 1.77(0.97-3.52) |
|  | Homemaker | 3.31(1.72-6.35) |
|  | Unemployed due to disability | 3.87(1.07-13.99) |
|  | Retired or unemployed for other reason | 0.97(0.65-1.46) |
| Femoral vein |  |  |
|  | Diameter (cm)≤0.65 | 6.35(1.16-103.97) |
|  | Mean reﬂux velocity (cm/s)＞8.2 | 7.82(1.45-130.3) |
|  | Peak reﬂux velocity (cm/s)＞24.5 | 25.77(10.56-331.12) |
|  | Reﬂux time (s)≤1.98 | 2.65(0.67-23.7) |
|  | Total reﬂuxed volume (mL)＞5.0 | 0.37(0.14-2.85) |
| History of recent surgery | yes | 1.86(0.8-3.8) |
| History of immobilization | yes | 1.55(0.8-2.93) |
| Popliteal vein |  |  |
|  | Diameter (cm)≤0.81 | 9.34(1.75-19.56) |
|  | Mean reﬂux velocity (cm/s)＞5.7 | 42.61(14.03-118.23) |
|  | Peak reﬂux velocity (cm/s)＞25.4 | 60.23(43.7-1238.97) |
|  | Reﬂux time (s)≤2.97 | 0.17(0.24-2.55) |
|  | Total reﬂuxed volume (mL)＞10.9 | 14.51(2.68-30.34) |
| Complete obstruction on ultrasound | yes | 1.19(0.51-2.43) |
| Compression compliance | >4 times/week | 0.41(0.18-0.86) |
| Calf muscle pump function at 6 weeks | ＜60% | 1.3(0.8-2) |
| Clot lysis time | Per 1 minute | 1.43(1.04-2.05) |
| Leptin | ＜1 ng/mL | 1.49(1.31-1.69) |
| Leg pain at Day 10 | per unit increment | 1.28(1.13-1.45) |
| iliac vein compression syndrome |  |  |
|  | Severe | 1.612(0.696-3.733) |
|  | Occlusion | 2.983(1.534-5.801) |
| More than one hypercoagable disorders | yes | 2.88(1.42-5.88) |
| Idiopathic DVT | yes | 0.9(0.5-1.4) |
| Concomitant antiplatelet/NSAID therapy | yes | 2.20(1.19-4.06) |
| Duration of compression therapy | <6mon | 2.894(1.595-5.251) |
| More extensive (less extensive Ref) DVT | More extensive DVT | 1.14(0.83-1.55) |
| Neutrophil-to-lymphocyte ratio | ≥ 2.6 | 2.35(1.06-5.18) |
| Overall hemostatic potential | OHP > 13.0 units | 2.17(1.06-4.43) |
| Reflux score at 6 weeks | ≥1 | 1.5(1-2.2) |
| Residual Femoral-Popliteal vein thrombosis | Yes | 1.881(0.987-3.586) |
| Residual Iliac-femoral vein thrombosis | Yes | 1.929(1.008-3.422) |
| Superficial reflux at 6 weeks | Yes | 1.6(1.1-2.3) |
| Tissue plasminogen activator | ≥1 ng/mL | 1.06(0.95-1.2) |
| Thrombosis score at 6 weeks | ≥1 | 2(1.6-3.5) |
| Thrombosis score _proximal_ at 6 weeks | ≥1 | 1.6(1.1-2.5) |
| Venous outflow resistance at 6 weeks venous outﬂow resistance | ≥1.5UR | 1.1(0.7-1.7) |
| α_2_-antiplasmin |  | 0.95(0.9-0.99) |
| Postnatal | Yes | 3.5(1.8-7) |
| Use of rivaroxaban on Day 10 (warfarin Ref) | Use of rivaroxaban | 0.53(0.33-0.86) |
| Ilio-femoral DVT | yes | 4.835(2.471-9.463) |
| Number of signs and symptoms |  | 1.329(1.171-1.524) |
