# Supplementary material for: A systematic evaluation and meta-analysis of early prediction of post-thrombotic syndrome
Source: Front Cardiovasc Med. 2023 Aug 24;10:1250480. doi: 10.3389/fcvm.2023.1250480 (PMC10484413; doi:10.3389/fcvm.2023.1250480)
Supplement: Supplementary file 3 [file Table3.docx]

**Table S1:** Analysis data of C-index

| Study | Data set | events | samplesize | FollowTime | Model | c-index | SE | low | up |
| --- | --- | --- | --- | --- | --- | --- | --- | --- | --- |
| Zhaoyu Wu2022(DT) | Traning set | 99 | 240 | 6m | DT | 0.722 | 0.030 | 0.591 | 0.854 |
| Tao Yu2022(GBDT) | Traning set | 92 | 555 | 24m | GBDT | 0.770 | 0.026 | 0.740 | 0.800 |
| Zhaoyu Wu2022(LR) | Traning set | 99 | 240 | 6m | LR | 0.814 | 0.026 | 0.699 | 0.928 |
| Jiantao Zhang2022(LR) | Traning set | 76 | 540 | 24m | LR | 0.825 | 0.026 | 0.774 | 0.877 |
| Tao Yu2022(LR) | Traning set | 92 | 555 | 24m | LR | 0.730 | 0.028 | 0.700 | 0.760 |
| Hao Huang2018(LR) | Traning set | 51 | 156 | 24m | LR | 0.825 | 0.034 | 0.747 | 0.903 |
| Peng Qiu2021(LR) | Traning set | 84 | 210 | 6m | LR | 0.713 | 0.033 | 0.648 | 0.778 |
| Anat Rabinovich2020(LR) | Traning set | 157 | 336 | 24m | LR | 0.700 | 0.025 | 0.640 | 0.770 |
| Elham E. Amin2018(LR) | Traning set | 236 | 451 | 24m | LR | 0.730 | 0.020 | 0.670 | 0.780 |
| Elham E. Amin2018(LR) | Traning set | 236 | 301 | 24m | LR | 0.620 | 0.024 | 0.550 | 0.680 |
| Zhaoyu Wu2022(RF) | Traning set | 99 | 240 | 6m | RF | 0.891 | 0.020 | 0.816 | 0.977 |
| Tao Yu2022(RF) | Traning set | 92 | 555 | 24m | RF | 0.810 | 0.024 | 0.780 | 0.840 |
| Zhaoyu Wu2022(XGBoost) | Traning set | 99 | 240 | 6m | XGBoost | 0.881 | 0.021 | 0.794 | 0.968 |
| Tao Yu2022(XGBoost) | Traning set | 92 | 555 | 24m | XGBoost | 0.770 | 0.026 | 0.740 | 0.800 |
| Tao Yu2022(GBDT) | Validation set | 17 | 117 | 24m | GBDT | 0.800 | 0.058 | 0.730 | 0.860 |
| Jiantao Zhang2022(LR) | Validation set | 40 | 268 | 24m | LR | 0.773 | 0.040 | 0.699 | 0.848 |
| Tao Yu2022(LR) | Validation set | 17 | 117 | 24m | LR | 0.830 | 0.054 | 0.760 | 0.890 |
| Hao Huang2018(LR) | Validation set | 45 | 135 | 24m | LR | 0.825 | 0.036 | 0.747 | 0.903 |
| Elham E. Amin2018(LR) | Validation set | 476 | 1107 | 24m | LR | 0.660 | 0.015 | 0.630 | 0.700 |
| Elham E. Amin2018(LR) | Validation set | 476 | 1107 | 24m | LR | 0.640 | 0.015 | 0.600 | 0.690 |
| A.Rabinovich2021(LR) | Validation set | 328 | 691 | 24m | LR | 0.630 | 0.019 | 0.590 | 0.670 |
| P. Prandoni2021(LR) | Validation set | 604 | 2349 | 36m | LR | 0.740 | 0.011 | 0.720 | 0.770 |
| Tao Yu2022(RF) | Validation set | 17 | 117 | 24m | RF | 0.760 | 0.062 | 0.660 | 0.840 |
| Tao Yu2022(XGBoost) | Validation set | 17 | 117 | 24m | XGBoost | 0.800 | 0.058 | 0.730 | 0.860 |

**Table S2:** Analysis data of Diagnostic Four Grid Table

| Study | events | samplesize | Follow time | Model | Sensitivity | Specificit | tp | fp | fn | tn |
| --- | --- | --- | --- | --- | --- | --- | --- | --- | --- | --- |
| Zhaoyu Wu2022(LR) | 99 | 240 | 6m | LR | 0.7365 | 0.6952 | 73 | 43 | 26 | 98 |
| Zhaoyu Wu2022(RF) | 99 | 240 | 6m | RF | 0.7740 | 0.8204 | 77 | 25 | 22 | 116 |
| Zhaoyu Wu2022(XGBoost) | 99 | 240 | 6m | XGBoost | 0.8473 | 0.7268 | 84 | 39 | 15 | 102 |
| Zhaoyu Wu2022(DT) | 99 | 240 | 6m | DT | 0.6330 | 0.8178 | 63 | 26 | 36 | 115 |
| Jiantao Zhang2022(LR) | 76 | 540 | 24m | LR | 0.7890 | 0.7090 | 60 | 135 | 16 | 329 |
| Tao Yu2022(LR) | 92 | 555 | 24m | LR | 0.6728 | 0.6214 | 62 | 175 | 30 | 288 |
| Tao Yu2022(RF) | 92 | 555 | 24m | RF | 0.7570 | 0.7019 | 70 | 138 | 22 | 325 |
| Tao Yu2022(XGBoost) | 92 | 555 | 24m | XGBoost | 0.6221 | 0.7740 | 57 | 105 | 35 | 358 |
| Tao Yu2022(GBDT) | 92 | 555 | 24m | GBDT | 0.7443 | 0.6475 | 68 | 163 | 24 | 300 |
| Peng Qiu2021(LR) | 84 | 210 | 6m | LR | 0.6190 | 0.7710 | 52 | 29 | 32 | 97 |
| Anat Rabinovich2020(LR) | 157 | 336 | 24m | LR | 0.1354 | 0.9118 | 21 | 16 | 136 | 163 |
| Elham E. Amin2018(LR) | 236 | 451 | 24m | LR | 0.6800 | 0.7160 | 160 | 61 | 76 | 154 |
| Elham E. Amin2018(LR) | 236 | 301 | 24m | LR | 0.9420 | 0.1800 | 222 | 53 | 14 | 12 |
| Jiantao Zhang2022(LR) | 40 | 268 | 24m | LR | 0.7250 | 0.6840 | 29 | 72 | 11 | 156 |
| Tao Yu2022(LR) | 17 | 117 | 24m | LR | 0.7533 | 0.7374 | 13 | 26 | 4 | 74 |
| Tao Yu2022(RF) | 17 | 117 | 24m | RF | 0.7316 | 0.7472 | 12 | 25 | 5 | 75 |
| Tao Yu2022(XGBoost) | 17 | 117 | 24m | XGBoost | 0.7873 | 0.6503 | 13 | 35 | 4 | 65 |
| Tao Yu2022(GBDT) | 17 | 117 | 24m | GBDT | 0.7914 | 0.6584 | 13 | 34 | 4 | 66 |
| P. Prandoni2021(LR) | 604 | 2349 | 36m | LR | 0.6600 | 0.7800 | 399 | 384 | 205 | 1361 |
